# Association of four metalloids in the serum and urine of individuals with major depressive disorders: a case–control study

**DOI:** 10.3389/fpsyt.2024.1403852

**Published:** 2024-06-12

**Authors:** Lei He, Shilong Li, Yan Huang, Yuxing Zhu, Lingzi Fan, Hongwei Zhang, Xiaofang Hou, Xiaoxin Li, Hongxin Deng, Xueli Guo, Chunxiao Liu, Chen Hu, Bing Cao

**Affiliations:** ^1^ Zhumadian Second People’s Hospital, Brain Hospital Affiliated to Zhengzhou University, Zhumadian, China; ^2^ Key Laboratory of Cognition and Personality, Faculty of Psychology, Ministry of Education, Southwest University, Chongqing, China

**Keywords:** metalloids, major depressive disorders, serum, urine, case-control

## Abstract

**Background:**

Major depressive disorder (MDD) pathogenesis may involve metalloids in a significant way. The aim of our study was to identify potential links between MDD and metalloid elements [boron (B), germanium (Ge), arsenic (As), antimony (Sb)].

**Methods:**

A total of 72 MDD cases and 75 healthy controls (HCs) were recruited from Zhumadian Second People’s Hospital in Henan Province, China. The levels of four metallic elements (B, Ge, As, and Sb) in the serum and urine were measured using inductively coupled plasma mass spectrometry (ICP-MS).

**Results:**

In comparison to the HCs, the B, As, and Sb levels were considerably lower in the MDD group (*p* < 0.05) in the serum; the MDD group had significantly higher (*p* < 0.05) and significantly lower (*p* < 0.001) B and Sb levels in the urine. After adjusting for potential confounders, serum B (OR = 0.120; 95% CI, 0.048, 0.300; *p* < 0.001) and Sb (OR = 0.133; 95% CI, 0.055, 0.322; *p* < 0.001) showed a negative correlation with MDD. Urine B had a negative correlation (OR = 0.393; 95% CI, 0.193, 0.801; *p* = 0.01) with MDD, while urine Sb had a positive correlation (OR = 3.335; 95% CI, 1.654, 6.726; *p* = 0.001) with MDD.

**Conclusion:**

Our current research offers insightful hints for future investigation into the function of metalloids in connection to MDD processes.

## 1 Introduction

Depression is one of the most common mental illnesses in modern society (1) and has affected more than 300 million individuals worldwide (2). The average lifetime prevalence of major depressive disorder (MDD) is projected to be 14.6% in high-income nations and 11.1% in low- and middle-income countries worldwide (3). A persistently depressed mood, together with symptoms like anxiety, agitation, sleeplessness, decreased appetite, and hallucinations, is the hallmark of MDD. In severe cases, some patients may even exhibit suicidal behaviors (4). For individuals, MDD carries the heaviest burden of disability among mental and behavioral disorders. Thus, MDD places a tremendous burden on sufferers, families, and society (5). According to projections by the World Health Organization, depression will account for the greatest global disease burden by 2030 (6).

Metalloids, which include boron (B), silicon (Si), germanium (Ge), arsenic (As), antimony (Sb), tellurium (Te), polonium (Po), and astatine (As), are elements that fall between metallic and non-metallic elements and have certain metallic and non-metallic properties. For these metalloids, the human body has relatively small concentrations of Te and At. The analysis of Si requires specific experimental conditions, while Po is a radioactive element that is not normally found in the human body. Thus, this paper primarily focuses on four metalloids, i.e., B, Ge, As, and Sb. Metalloids play important roles in maintaining the physical and mental health of the human body (7). For example, low B concentrations have been linked to immunological dysfunction, osteoporosis, cognitive decline, and an increased risk of death (7). Several studies have stated that autism is associated with the dysregulation of Ge in the serum and urine (8). Furthermore, peripheral neuropathy and impaired sensory function were reported to be associated with high concentrations of As in human urine (9). In a case–control study, an increased risk of developing attention deficit disorder was associated with dimethylarsinic acid (DMAV, low) and urinary levels of As (high) (10). Many additional pieces of research also suggest a strong correlation between changes in As and mental health (11). With regard to Sb, one of the metalloid elements, its levels in the urine are linked to cognitive function, according to an intentional study of older Americans (12). The concentration of Sb in urine was reported to be positively correlated with the symptoms of MDD (13).

Currently, the available data on the processes between metalloid elements and MDD are limited. Additionally, there are only a few research studies that have thoroughly investigated the association between the levels of metalloid elements in blood and urine and MDD. To further examine the role of metalloids in the pathophysiology of MDD, our study analyzed the levels of four specific metalloids (namely, B, Ge, As, Sb) in patients diagnosed with MDD and a control group of healthy adults (HCs). The current study investigated the association between MDD and the levels of four metalloids in serum and urine, which could provide a comprehensive understanding of metalloid profiles in MDD individuals. This approach adds new research evidence by simultaneously measuring multiple metalloids in both serum and urine samples within a single study. By uncovering potential correlations and distinct concentration patterns, our research sheds new light on the relationship between metalloid exposure and MDD pathogenesis. This provides vital information for future studies on identifying biomarkers and developing therapeutic approaches.

## 2 Methods

### 2.1 Ethical approval

The study protocols have undergone scrutiny and received approval from the Medical Ethics Committee of Zhumadian Second People’s Hospital in Henan Province (Approval no. IRB-2021–006-02). Prior to their participation in the study, the MDD cases and HCs signed a written informed consent form. All procedures adhered to the Helsinki Declaration standards.

### 2.2 Study population

A total of 72 persons diagnosed with MDD and 75 healthy participants from Zhumadian Second People’s Hospital in Henan Province were selected as the case and control group, respectively. The inclusion criteria were as follows: 1) must meet the criteria outlined in the Diagnostic and Statistical Manual of Mental Disorders, 5th edition (DSM-5); 2) must not have taken antidepressants or antipsychotics within the past month; 3) must have an education level at or above primary school; 4) Hamilton Depression Scale (HAMD) - 24 version score ≥20; and 5) must be between the ages of 18 and 60, with no sex restrictions. The inclusion criteria of HCs were as follows: 1) must match the sex, age, and place of residence of the case group; 2) must have an education level at or above primary school; and 3) routine hematology, urine and feces, liver function, fasting blood glucose, renal function, chest X-ray, electrocardiogram, etc. are normal.

The exclusion criteria of the HC group are similar to the MDD group. The exclusion criteria for all participants are as follows: 1) previous occurrence of organic brain disease or diagnosed neurologic disorder (e.g., Parkinson’s, cerebral hemorrhage, massive cerebral infarction, encephalitis, epilepsy); 2) presence of severe medical conditions that are clinically significant or unstable, including the liver, kidneys, gastrointestinal system, respiratory system, cardiovascular system, endocrine system, blood, neurological system, genitourinary system, musculoskeletal system, or metabolic-related issues; 3) intellectual disabilities; 4) history of alcohol, drugs, chemicals, substances, or psychoactive substance abuse; 5) visual or auditory impairments; and 6) currently pregnant or breastfeeding.

### 2.3 Basic and clinical information collection

This study collected general information about all participants, including their sex, age, education level, drinking and smoking status, body mass index, and marital status. The degree of pleasure deficit was measured using the Snaith-Hamilton Pleasure Scale (SHAPS), while the physical and mental health index was measured using the World Health Organization-Five Well-Being Index (WHO-5). The HAMD-24 was employed to assess the severity of psychiatric symptoms, while the Clinical Global Impression Severity (CGI-S) and Improvement (CGI-I) scores were utilized to appraise particular symptom domains linked to MDD.

### 2.4 Serum and urinary sample preparation and measurement

Blood samples of approximately 8.5 mL were collected from fasting individuals during the morning hours (7–9 a.m.) on the following day. The blood was extracted using venipuncture and placed into gel-separator tubes to obtain serum. Following coagulation for 1 h at 4°C, the upper layer of the serum was isolated by centrifugation at 3,000×*g* for 10 min at 4°C. The separated serum samples were promptly transferred into cryovials with a volume of 5 mL. A urine collection tube was utilized to gather approximately 10 mL of urine in the morning. The samples were stored at a temperature of −80°C for further examination of metalloids.

The operations of the experiment were as follows: taking 0.1 mL of serum (or urinary) sample into a 2-mL centrifuge tube, putting 0.1 mL of combined internal standard indium (In) and rhenium (Re) and adding 1% nitric acid, shaking well, and then measuring on a machine. We used Perkin-Elmer Sciex’s Elan DRC II inductively coupled plasma mass spectrometer (ICP-MS) and Agilent’s 7700x ICP-MS to analyze the metalloids.

### 2.5 Statistical analysis

Descriptive statistics were performed, with continuous variables summarized as the mean and standard deviation (SD) or median and interquartile range (IQR) and categorical variables summarized as frequencies and proportions. The independent samples *t*-test was used to compare the differences between the two groups for normally distributed continuous variables. The Mann–Whitney *U* test was used to compare the differences between the two groups for non-normally distributed continuous variables. Due to the non-normal distribution of the metalloids’ concentrations, the concentrations of all metalloids were represented by the median along with the upper and lower quartiles (P25–P75). Unconditional logistic regression models were used to explore the relationships between patients and healthy controls and the metalloid levels. The median value of the metalloid concentrations in HCs was used as the cutoff value in the logistic regression analysis. Odds ratios (ORs), *β*-values, and their 95% confidence intervals (CIs) were estimated using maximum likelihood methods. The variables of age, sex, education level, smoking status, alcohol consumption, and BMI were adjusted in the logistic regression models. Partial correlation analysis on ranks (i.e., Spearman correlation) was used to calculate the correlation coefficients among metalloid levels in the serum and urine as well as metalloid levels with clinical variables, including HAMD-24, SHAPS, WHO-5, CGI-S, and CGI-I. A two-sided *p <*0.05 was statistically significant. All statistical analysis was performed using SPSS 28.0 (SPSS Inc., Chicago, IL, USA).

## 3 Results

### 3.1 Demographic characteristics of the subjects

A total of 147 individuals were included in the analysis, consisting of 72 patients with MDDs and 75 HCs. The mean age (SD) of the MDD and HC groups were 39.3 ± 15.5 and 41.9 ± 6.9 years, respectively. The demographic characteristics, including age, sex, education level, and the number of smokers and drinkers, were comparable in the two groups, and there were no statistically significant differences observed (*p* > 0.05). The BMI showed a statistically significant difference between the two groups (MDDs: 22.2 ± 4.3 kg/m^2^; HCs: 23.9 ± 2.10 kg/m^2^; *p* = 0.004). The scores on the HAMD-24, SHAPS, and WHO-5 scales within the MDDs were significantly higher in comparison to those observed in the HCs (HAMD-24: 26.8 ± 6.0 vs. 2.1 ± 1.5, *p* < 0.001; SHAPS: 38.1 ± 5.2 vs. 18.5 ± 3.7, *p* < 0.001; WHO-5: 22.2 ± 6.0 vs. 7.1 ± 2.4, *p* < 0.001). The demographic and clinical characteristics of the MDD and HC groups are shown in **Table 1**.

**Table 1:** Demographic characteristics of the included participants.

Variable	MDD (*n* = 72)	HC (*n* = 75)	*t*/χ^2^	*p*-values
**Age, years, mean ± SD**	39.3 ± 15.5	41.9 ± 6.9	1.354	0.178
**Sex (female/male); *n*/%**	36/36 (50.0/50.0)	49/26 (65.3/34.7)	3.541	0.060
**Education level; *n*/%**			0.964	0.810
Primary school or below	14 (19.4)	17 (22.7)		
Middle school	32 (44.4)	35 (46.7)		
High school	17 (23.6)	17 (22.7)		
Graduate or above	9 (12.5)	6 (8.0)		
**Drink alcohol (yes/no), *n*/%**	9/63 (12.5/87.5)	15/60 (20.0/80.0)	1.513	0.219
**Smoke cigarettes (yes/no), *n*/%**	6/66 (8.3/91.7)	14/61 (18.7/81.3)	3.337	0.068
**HAMD-17 total score, mean ± SD**	26.8 ± 6.0	2.1 ± 1.5	−34.649	<0.001
**SHAPS score, mean ± SD**	38.1 ± 5.2	18.5 ± 3.7	−26.487	<0.001
**WHO-5 score, mean ± SD**	22.2 ± 6.0	7.1 ± 2.4	−20.471	<0.001
**BMI, kg/m^2^, mean ± SD**	22.2 ± 4.3	23.9 ± 2.1	2.937	0.004

### 3.2 The levels of metalloids in the serum and urine of the MDD and HC groups

The distributions and comparisons of the four metalloid levels in the serum and urine of the MDD and HC groups are shown in **Table 2**. The results of the independent-samples Mann–Whitney *U* test indicated that the levels of B in the serum were significantly lower in MDDs compared to HCs (cases: 16.29 ng/mL, IQR: 12.29–21.87 ng/mL; HCs: 30.35 ng/mL, IQR: 23.78–39.39 ng/mL; *Z* = −7.405, *p* < 0.001), the levels of As in the serum were significantly lower in MDDs compared to HCs (cases: 0.27 ng/mL, IQR: 0.16–0.37 ng/mL; HCs: 0.33 ng/mL, IQR: 0.24–0.48 ng/mL; *Z* = −2.923, *p* < 0.05), and the levels of Sb in the serum were significantly lower in MDDs compared to HCs (cases: 3.06 ng/mL, IQR: 2.73–3.66 ng/mL; HCs: 4.29 ng/mL, IQR: 3.47–4.99 ng/mL; *Z* = −5.846, *p* < 0.001). The levels of B in the urine were also significantly lower in MDDs when compared with HCs (cases: 618.68 ng/mL, IQR: 382.34–1,040.84 ng/mL; HCs: 1,050.55 ng/mL, IQR: 774.48–1,782.16 ng/mL; *Z* = −5.125, *p* < 0.001). Moreover, the levels of Sb in the urine were also significantly higher in MDDs compared with HCs (cases: 0.18 ng/mL, IQR: 0.10–0.27 ng/mL; HCs: 0.10 ng/mL, IQR: 0.06–0.19 ng/mL; *Z* = 3.332, *p* < 0.05).

**Table 2:** The concentrations of metalloids in the serum or urine between the MDD cases and HCs.

Metalloids	MDD (*n* = 72)	HC (*n* = 75)	*Z*	*p-*values
Serum				
B (ng/ mL, IQR)	16.29 (12.29, 21.87)	30.35 (23.78, 39.39)	−7.405	<0.0001
Ge (ng/ mL, IQR)	1.36 (0.83, 1.99)	1.57 (1.05, 2.27)	−1.624	0.104
As (ng/ mL, IQR)	0.27 (0.16, 0.37)	0.33 (0.24, 0.48)	−2.923	0.003
Sb (ng/ mL, IQR)	3.06 (2.73, 3.66)	4.29 (3.47, 4.99)	−5.846	<0.0001
Urine				
B (ng/ mL, IQR)	618.68 (382.34, 1,040.84)	1,050.55 (774.48, 1,782.16)	−5.125	<0.0001
Ge (ng/ mL, IQR)	0.85 (0.62, 1.17)	0.86 (0.60, 1.06)	−0.229	0.818
As (ng/ mL, IQR)	16.03 (10.48, 18.96)	15.66 (10.94, 19.23)	0.426	0.670
Sb (ng/ mL, IQR)	0.18 (0.10, 0.27)	0.10 (0.06, 0.19)	3.332	0.001

Unit: ng/mL serum.

IQR, interquartile range.

### 3.3 Logistic regression analysis of the association between metalloids and MDD in the serum and urine

The results of logistic regression are presented in **Table 3**. Univariate analyses revealed that elevated serum levels of B and Sb were significantly associated with MDD. Significance was maintained after adjusting the potential confounders of age, sex, education level, smoking status, alcohol consumption, and BMI. The adjusted OR revealed that serum levels of B (OR = 0.120; 95% CI, 0.048, 0.300; *p* < 0.001) were negatively associated with MDD and that elevated serum levels of Sb (OR = 0.133; 95% CI, 0.055, 0.322; *p* < 0.001) were associated with MDD. Univariate analyses also revealed that elevated urine levels of B were significantly associated with MDD. Significance was maintained after adjusting the potential confounders of age, sex, education level, smoking status, alcohol consumption, and BMI. The adjusted OR revealed that urine levels of B (OR = 0.393; 95% CI, 0.193, 0.801; *p* = 0.05) were negatively associated with MDD, and the adjusted OR revealed that urine levels of Sb (OR = 3.335; 95% CI, 1.654, 6.726; *p* = 0.001) were positively associated with MDD (**Table 3**).

**Table 3:** Logistic regression analysis of serum and urinary concentrations of metalloids in MDD cases and HCs.

Metalloids	Group	MDD	HC	UOR^a^ (95% CI)	*p-*values	AOR^b^ (95% CI)	*p-*values
Serum							
**B**	Low	65 (90.3)	38 (50.7)	0.189 (0.084, 0.424)	<0.001	0.120 (0.048, 0.300)	<0.001
	High	7 (9.7)	37 (50.3)				
**Ge**	Low	45 (62.5)	37 (50.3)	0.711 (0.434, 1.164)	0.175	0.612 (0.304, 1.230)	0.168
	High	27 (37.5)	38 (50.7)				
**As**	Low	45 (62.5)	37 (50.3)	0.711 (0.434, 1.164)	0.175	0.692 (0.355, 1.347)	0.278
	High	27 (37.5)	38 (50.7)				
**Sb**	Low	63 (87.5)	37 (50.3)	0.237 (0.115, 0.49)	<0.001	0.133 (0.055, 0.322)	<0.001
	High	9 (12.5)	38 (50.7)				
Urine							
**B**	Low	54 (75.0)	37 (50.3)	0.486 (0.277, 0.854)	0.012	0.393 (0.193, 0.801)	0.010
	High	18 (25.0)	38 (50.7)				
**Ge**	Low	35 (48.6)	38 (50.7)	0.974 (0.619, 1.531)	0.908	1.189 (0.612, 2.312)	0.609
	High	37 (51.4)	37 (50.3)				
**As**	Low	31 (43.1)	38 (50.7)	1.108 (0.711, 1.728)	0.651	1.609 (0.825, 3.14)	0.163
	High	41 (56.9)	37 (50.3)				
**Sb**	Low	19 (26.4)	38 (50.7)	1.432 (0.941, 2.18)	0.093	3.335 (1.654, 6.726)	0.001
	High	53 (73.6)	37 (50.3)				

a Determined by an unconditional logistic regression model.

b Adjusted OR and 95% CI were determined by an unconditional logistic regression model after adjusting for age, sex, education level, smoking status, alcohol consumption, and BMI.

### 3.4 The element correlations between serum metalloid levels and urine levels within MDDs and HCs

**Figure 1:**
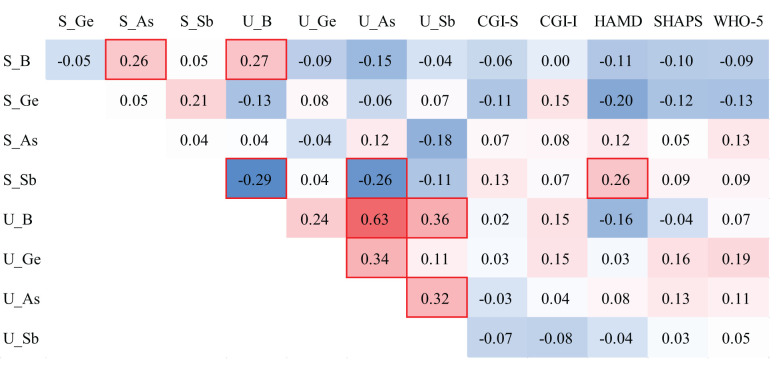
Spearman correlations of metalloid levels in the serum and urine of individuals with MDD. The red background represents the positive correlations between the two compared variables, while the blue background represents the negative correlations. The red box indicates statistical significance.

In the correlation analyses of metalloids and clinical variables, we only found that serum Sb levels were positively correlated with HAMD scores (*r* = 0.26). Regarding the correlation between metalloids in the serum and urine, we observed that serum B was positively correlated with serum As levels (*r* = 0.26), serum B was positively correlated with urinary B levels (*r* = 0.27), serum Sb levels were negatively correlated with urinary B levels (*r* = −0.29), and serum Sb levels were negatively correlated with urinary As levels (*r* = −0.26). In urine, B content was positively correlated with urinary As content (*r* = 0.63), B content was positively correlated with urinary Sb content (*r* = 0.36), Ge content was positively correlated with urinary As content (*r* = 0.34), and As content was positively correlated with urinary Sb content (*r* = 0.32) (**Figure 1**).

## 4 Discussion

This study conducted a systematic investigation on the levels of four metalloids (namely, B, Ge, As, and Sb) in serum and urine samples from a total of 147 individuals with MDD and healthy controls. Lower levels of B were observed both in serum and urine samples of MDD, whereas lower serum Sb levels but higher urine Sb levels were reported in individuals with MDD. While a notable decrease in the amount of As was found in the serum of persons with MDD, the findings of the logistic analysis indicated that there was no significant association between Ge and As with MDD. Our findings suggest that the dysregulation of B and Sb may play a role in the onset and advancement of MDD.

As a prebiotic chemical element, B has played an important role in the origin and evolution of life (14). Although B has not been classified as an essential micronutrient for humans and animals, it has a positive impact on various biological activities, including brain function, immunity, and the production of steroid hormones like estrogen (15). It is important to maintain certain B levels in the body to ensure the functioning of the immune system and bone health (15–18). It has also been shown that the levels of B in the serum and urine are negatively correlated with the levels of thyroid hormones in the body, and experimental studies have also indicated that B has a significant influence on the hormone levels of the thyroid gland (19). There is a potential association between thyroid and depression (20, 21). Thus, it is plausible to speculate that there might also be a connection between B and depression. The results of this experiment indicate that MDD patients have significantly lower levels of B in the serum and urine than healthy individuals. It can be hypothesized that increasing the level of B in the body may help alleviate depressive symptoms. However, limited research specifically investigates this relationship, and further exploration is warranted based on previous studies and our findings.

Sb is a metalloid extensively utilized in industrial manufacturing (13). Our research discovered an association between Sb levels and MDD, and serum Sb levels were reported to be positively correlated with HAMD-24 scores. Hence, it may be inferred that there exists a link between the levels of Sb and the severity of MDD. Consistent with our findings, several studies have demonstrated a positive association between increased levels of Sb in urine and the risk of death from cardiovascular disease (13). Existing evidence supported that Sb has the potential to induce Alzheimer’s disease in mice (22, 23), which can explain its neurotoxic effects. Meanwhile, Alzheimer’s disease shares numerous similarities with depression (24). A recent study by Zhang et al. found a noteworthy positive association between urine Sb levels and depressive symptoms in the general population, which revealed a substantial correlation between higher urinary Sb concentrations and increased depressed symptoms (13). These findings align with the outcomes of the current study. The urinary Sb concentrations were markedly elevated in individuals with MDDs compared to HCs. Interestingly, we found that serum Sb concentrations were significantly lower in MDDs than in HCs. It is possible to speculate that a widespread disruption of Sb metabolism occurs in individuals with MDD.

The findings from our study indicate that Ge and As might not be significantly associated with MDD. To the best of our knowledge, there is a lack of research specifically focusing on the relationship between Ge and MDD. As, a metalloid commonly found in groundwater, has been identified as a nuisance material that can impact human health (11). Previous evidence demonstrated that residents of As-contaminated areas experience a higher incidence of mental health issues (25). Additionally, long-term exposure to As has been linked to a decline in cognitive function in adults and can disrupt the molecular mechanisms of the central nervous system (25). Although we found significantly lower levels of As in individuals with MDDs than HCs, this difference did not hold when considering logistic regression. These results contradict previous studies, suggesting that the influence of As on MDD may be indirect and mediated through other mechanisms.

It is usual to observe correlations between different trace elements within the human body. Furthermore, the interactions among trace elements in diseases, including depression, have been extensively explored. Trace elements may exhibit synergistic or antagonistic associations. For example, studies have shown that increased zinc intake can impede the absorption of copper into the bloodstream (26). One study using isotope tracing methods found that a high-iron diet resulted in a 60% decrease in copper absorption in the intestines of mice (27). Trace elements also exhibit indirect competitive inhibition. High levels of calcium supplementation greatly decrease the absorption of iron in the intestines. Notably, the inhibitory effect of calcium on iron absorption is related to the form of calcium present (28). In a poultry experiment, high concentrations of copper supplemented through the digestive tract decreased the absorption rates of zinc and calcium by intestinal cells (29). Previous research studies have not explicitly documented the factors contributing to the correlation between metalloids and MDD. From the above information provided, we hypothesize that there may be a direct or indirect synergistic or antagonistic connection among metalloids in MDD.

Furthermore, our study found distinct different changes in the levels of metalloids in the serum and urine of individuals with MDD as compared to the HC group. The reason for this is the serum and urine are indicative of distinct metabolic states within the human body. The metabolism of serum primarily focuses on various biomarkers present in the serum, which can be either internally generated or acquired from the surroundings (30). Urine is a terminal metabolic product generated through the filtration of blood by the glomeruli, followed by reabsorption, excretion, and secretion by the renal tubules and collecting ducts. Modifications in the composition, quantity, and properties of urine can reflect the overall metabolic state of the body (31). When compared to serum, urine samples are non-invasive to obtain, convenient to collect, and have a relatively simple protein composition, making them easier to analyze. The differences in the observed outcomes may be attributed to the distinct biomarkers present in the serum and urine.

### 4.1 Limitations

Several limitations should be addressed. Firstly, while metalloids were quantified in the serum and urine in the current study, it is possible that metalloids present in other body components such as the nails and hair could potentially be linked to mental diseases, including depression. Secondly, due to the nature of being a cross-sectional study, establishing a cause-and-effect relationship is challenging in this particular study. Thirdly, some basic information, such as BMI, was statistically different between the two comparison groups, which may be related to the selection of study samples. Fourthly, our study did not investigate the nutritional supplements and dietary status of the study subjects, which could have a potential influence on metalloid levels in the body. Furthermore, it is crucial to acknowledge that MDD patients with scores exceeding 20 on the HAMD-24 were classified as having moderate depression, which could potentially impact appetite, food intake, and water intake. This could introduce confounding factors that may have influenced the outcomes of our study.

## 5 Conclusion

In conclusion, our findings suggest that individuals with MDD have lower serum and urine levels of B, while they have lower serum levels of Sb but higher urinary levels of Sb. It is possible to hypothesize that altering the metabolism of B or Sb in the human body could potentially be associated with the symptoms of MDD. Additional developmental and toxicological investigations should be carried out in animal models to clarify the underlying mechanisms of the associations observed in this study. Further validation is needed to determine the involvement of elements B and Sb in MDD, particularly through longitudinal investigations and animal experiments.

## Data availability statement

The raw data supporting the conclusions of this article will be made available by the authors, without undue reservation.

## Ethics statement

The studies involving humans were approved by the Medical Ethics Committee of Zhumadian Second People’s Hospital in Henan Province. The studies were conducted in accordance with the local legislation and institutional requirements. Written informed consent for participation in this study was provided by the participants’ legal guardians/next of kin.

## Author contributions

LH: Writing – original draft, Investigation, Formal analysis, Data curation. SL: Writing – original draft, Software, Investigation, Data curation. YH: Writing – review & editing, Writing – original draft, Methodology. YZ: Writing – review & editing, Investigation. LF: Writing – review & editing, Investigation. HZ: Writing – review & editing, Investigation. XH: Writing – review & editing, Validation, Investigation. XL: Writing – review & editing, Methodology, Investigation. HD: Writing – review & editing, Investigation. XG: Writing – review & editing, Investigation. CL: Writing – review & editing, Software, Formal analysis. CH: Writing – review & editing, Investigation. BC: Writing – review & editing, Supervision, Methodology, Formal analysis.

## References

[1] Li Y Cheng Y Zhou Y Du H Zhang C Zhao Z. High fat diet-induced obesity leads to depressive and anxiety-like behaviors in mice via AMPK/mTOR-mediated autophagy. Exp Neurol. (2022) 348:113949. doi: 10.1016/j.expneurol.2021.113949 34902357

[2] Davis AK Barrett FS May DG Cosimano MP Sepeda ND Johnson MW. Effects of psilocybin-assisted therapy on major depressive disorder: A randomized clinical trial. JAMA Psychiatry. (2021) 78:481. doi: 10.1001/jamapsychiatry.2020.3285 33146667 PMC7643046

[3] Auerbach RP Mortier P Bruffaerts R Alonso J Benjet C Cuijpers P. WHO World Mental Health Surveys International College Student Project: Prevalence and distribution of mental disorders. J Abnormal Psychol. (2018) 127:623–38. doi: 10.1037/abn0000362 PMC6193834 30211576

[4] Belvederi Murri M Amore M Respino M Alexopoulos GS. The symptom network structure of depressive symptoms in late-life: Results from a European population study. Mol Psychiatry. (2020) 25:1447–56. doi: 10.1038/s41380-018-0232-0 30171210

[5] Bromet E Andrade LH Hwang I Sampson NA Alonso J De Girolamo G. Cross-national epidemiology of DSM-IV major depressive episode. BMC Med. (2011) 9:90. doi: 10.1186/1741-7015-9-90 21791035 PMC3163615

[6] Kupfer DJ Frank E Phillips ML. Major depressive disorder: new clinical, neurobiological, and treatment perspectives. Focus. (2016) 14:266–76. doi: 10.1176/appi.focus.140208 PMC6519641 31997953

[7] Khaliq H Juming Z Ke-Mei P. The physiological role of boron on health. Biol Trace Elem Res. (2018) 186:31–51. doi: 10.1007/s12011-018-1284-3 29546541

[8] Blaurock-Busch E Amin OR Rabah T. Heavy metals and trace elements in hair and urine of a sample of Arab children with autistic spectrum disorder. Mædica. (2011) 6:247–57. doi: 10.1016/S0924-9338(12)74420-6 PMC3391939 22879836

[9] Hafeman DM Ahsan H Louis ED Siddique AB Slavkovich V Cheng Z. Association between arsenic exposure and a measure of subclinical sensory neuropathy in Bangladesh. J Occup Environ Med. (2005) 47:778–84. doi: 10.1097/01.jom.0000169089.54549.db 16093927

[10] Yang Y-W Liou S-H Hsueh Y-M Lyu W-S Liu C-S Liu H-J. Risk of Alzheimer’s disease with metal concentrations in whole blood and urine: A case–control study using propensity score matching. Toxicol Appl Pharmacol. (2018) 356:8–14. doi: 10.1016/j.taap.2018.07.015 30025849

[11] Garza-Lombó C Pappa A Panayiotidis MI Gonsebatt ME Franco R. Arsenic-induced neurotoxicity: A mechanistic appraisal. J Biol Inorgan Chem: JBIC: A Publ Soc Biol Inorgan Chem. (2019) 24:1305–16. doi: 10.1007/s00775-019-01740-8 PMC6903391 31748979

[12] Shiue I. People with diabetes, respiratory, liver or mental disorders, higher urinary antimony, bisphenol A, or pesticides had higher food insecurity: USA NHANES 2005–2006. Environ Sci Pollut Res Int. (2016) 23:198–205. doi: 10.1007/s11356-015-5677-y 26517997

[13] Zhang T Luo J Ge H Hao K Wang Z Zhang D. Relationships between urinary antimony concentrations and depressive symptoms in adults. Chemosphere. (2022) 291:133104. doi: 10.1016/j.chemosphere.2021.133104 34856240

[14] Biţă A Scorei IR Bălşeanu TA Ciocîlteu MV Bejenaru C Radu A. New insights into boron essentiality in humans and animals. Int J Mol Sci. (2022) 23:9147. doi: 10.3390/ijms23169147 36012416 PMC9409115

[15] Devirian TA Volpe SL. The physiological effects of dietary boron. Crit Rev Food Sci Nutr. 43(2):219–31. doi: 10.1080/10408690390826491 12705642

[16] Donoiu I Militaru C Obleagă O Hunter JM Neamţu J Biţă A. Effects of boron-containing compounds on cardiovascular disease risk factors – A review. J Trace Elem Med Biol. (2018) 50:47–56. doi: 10.1016/j.jtemb.2018.06.003 30262316

[17] Penland JG. The importance of boron nutrition for brain and psychological function. Biol Trace Elem Res. (1998) 66:299–317. doi: 10.1007/BF02783144 10050926

[18] Scorei R. Is boron a prebiotic element? A mini-review of the essentiality of boron for the appearance of life on earth. Orig Life Evol Biosphere. (2012) 42:3–17. doi: 10.1007/s11084-012-9269-2 22528885

[19] Ev P Aa T Op A Mg S Av S. Boron—A potential goiterogen? Med Hypotheses. (2017) 104:63–7. doi: 10.1016/j.mehy.2017.05.024 28673593

[20] Costantine MM Smith K Thom EA Casey BM Peaceman AM Varner MW. Effect of thyroxine therapy on depressive symptoms among women with subclinical hypothyroidism. Obstet Gyneco. (2020) 135:812–20. doi: 10.1097/AOG.0000000000003724 PMC7103482 32168208

[21] Karakatsoulis GN Tsapakis E-M Mitkani C Fountoulakis KN. Subclinical thyroid dysfunction and major depressive disorder. Hormones (Athens Greece). (2021) 20:613–21. doi: 10.1007/s42000-021-00312-3 34427900

[22] Wang X Zhu P Xu S Liu Y Jin Y Yu S. Antimony, a novel nerve poison, triggers neuronal autophagic death via reactive oxygen species-mediated inhibition of the protein kinase B/mammalian target of rapamycin pathway. Int J Biochem Cell Biol. (2019) 114:105561. doi: 10.1016/j.biocel.2019.105561 31228582

[23] Xu S Yang Z Zhi Y Yu S Zhang T Jiang J. The effects of antimony on Alzheimer’s disease-like pathological changes in mice brain. Sci Total Environ. (2021) 760:143235. doi: 10.1016/j.scitotenv.2020.143235 33183805

[24] Galts CPC Bettio LEB Jewett DC Yang CC Brocardo PS Rodrigues ALS. Depression in neurodegenerative diseases: Common mechanisms and current treatment options. Neurosci Biobehav Rev. (2019) 102:56–84. doi: 10.1016/j.neubiorev.2019.04.002 30995512

[25] Karim Y Siddique AE Hossen F Rahman M Mondal V Banna HU. Dose-dependent relationships between chronic arsenic exposure and cognitive impairment and serum brain-derived neurotrophic factor. Environ Int. (2019) 131:105029. doi: 10.1016/j.envint.2019.105029 31352261

[26] Tezvergil-Mutluay A Carvalho RM Pashley DH. Hyperzincemia from ingestion of denture adhesives. J Prosthet Dent. (2010) 103:380–3. doi: 10.1016/S0022-3913(10)60081-9 20493327

[27] Wang T Xiang P Ha J-H Wang X Doguer C Flores SRL. Copper supplementation reverses dietary iron overload-induced pathologies in mice. J Nutr Biochem. (2018) 59:56–63. doi: 10.1016/j.jnutbio.2018.05.006 29960117 PMC6467079

[28] Candia V Rios-Castillo I Carrera-Gil F Vizcarra B Olivares M Chaniotakis S. Effect of various calcium salts on non-heme iron bioavailability in fasted women of childbearing age. J Trace Elem Med Biol. (2018) 49:8–12. doi: 10.1016/j.jtemb.2018.04.029 29895376

[29] Ognik K Stepniowska A Cholewinska E Kozlowski K. The effect of administration of copper nanoparticles to chickens in drinking water on estimated intestinal absorption of iron, zinc, and calcium. Poultry Sci. (2016) 95:2045–51. doi: 10.3382/ps/pew200 27307476

[30] Bar N Korem T Weissbrod O Zeevi D Rothschild D Leviatan S. A reference map of potential determinants for the human serum metabolome. NATURE. (2020) 588:135–40. doi: 10.1038/s41586-020-2896-2 33177712

[31] Wu Q Fenton RA. Urinary proteomics for kidney dysfunction: Insights and trends. Expert Rev Proteomics. (2021) 18:437–52. doi: 10.1080/14789450.2021.1950535 34187288

